# Quantitative Systems Pharmacology Model-Based Predictions of Clinical Endpoints to Optimize Warfarin and Rivaroxaban Anti-Thrombosis Therapy

**DOI:** 10.3389/fphar.2020.01041

**Published:** 2020-07-14

**Authors:** Sonja Hartmann, Konstantinos Biliouris, Lawrence J. Lesko, Ulrike Nowak-Göttl, Mirjam N. Trame

**Affiliations:** ^1^ Center for Pharmacometrics & Systems Pharmacology, Department of Pharmaceutics, University of Florida, Orlando, FL, United States; ^2^ Thrombosis & Hemostasis Treatment Center, Institute of Clinical Chemistry, University of Schleswig-Holstein, Germany

**Keywords:** anticoagulation network, biomarker, quantitative systems pharmacology, rivaroxaban, warfarin, precision dosing

## Abstract

**Background:**

Tight monitoring of efficacy and safety of anticoagulants such as warfarin is imperative to optimize the benefit-risk ratio of anticoagulants in patients. The standard tests used are measurements of prothrombin time (PT), usually expressed as international normalized ratio (INR), and activated partial thromboplastin time (aPTT).

**Objective:**

To leverage a previously developed quantitative systems pharmacology (QSP) model of the human coagulation network to predict INR and aPTT for warfarin and rivaroxaban, respectively.

**Methods:**

A modeling and simulation approach was used to predict INR and aPTT measurements of patients receiving steady-state anticoagulation therapy. A previously developed QSP model was leveraged for the present analysis. The effect of genetic polymorphisms known to influence dose response of warfarin (*CYP2C9, VKORC1*) were implemented into the model by modifying warfarin clearance (CYP2C9 *1: 0.2 L/h; *2: 0.14 L/h, *3: 0.04 L/h) and the concentration of available vitamin K (VKORC1 GA: −22% from normal vitamin K concentration; AA: −44% from normal vitamin K concentration). Virtual patient populations were used to assess the ability of the model to accurately predict routine INR and aPTT measurements from patients under long-term anticoagulant therapy.

**Results:**

The introduced model accurately described the observed INR of patients receiving long-term warfarin treatment. The model was able to demonstrate the influence of genetic polymorphisms of *CYP2C9* and *VKORC1* on the INR measurements. Additionally, the model was successfully used to predict aPTT measurements for patients receiving long-term rivaroxaban therapy.

**Conclusion:**

The QSP model accurately predicted INR and aPTT measurements observed during routine therapeutic drug monitoring. This is an exemplar of how a QSP model can be adapted and used as a model-based precision dosing tool during clinical practice and drug development to predict efficacy and safety of anticoagulants to ultimately help optimize anti-thrombotic therapy.

## Highlights

- Anticoagulant therapy remains challenging due to the influence of genetic factors
- Modeling and Simulation is a valuable tool to help understand complex systems and optimize therapy
- Developed model accurately predicts INR following warfarin administration for patients with different genetic polymorphisms
- In addition, the model accurately predicts aPTT for patients under steady-state rivaroxaban treatment
- The developed model may be used to optimize treatments for patients requiring anticoagulant therapy

## Introduction

Venous thromboembolism (VTE) is a common cardiovascular disease, associated with a high morbidity and mortality rate (Naess et al., 2007; Beckman et al., 2010). The gold standard of care for VTE is warfarin, making it the most commonly prescribed anticoagulant currently on the market (Garcia and Spyropoulos, 2008). Warfarin acts *via* the inhibition of the vitamin K epoxide reductase (VKOR) within the vitamin K (VK) cycle. It effectively inhibits the production of all VK-dependent coagulation factors, namely factors II, VII, IX, X, protein C (PC), and protein S (PS) (Thompson, 1977; Weiss et al., 1987; Zivelin et al., 1993).

Several alternatives to warfarin have entered the market over the past years. Especially the class of direct oral anticoagulants (DOACs) has reached increasing recognition as substitute for warfarin, with potentially less side effects (Levy et al., 2014; Van Es et al., 2014), which generally exert their effects further downstream in the clotting cascade.

Tight monitoring of anticoagulants is imperative, especially the older generation of anticoagulants (i.e. vitamin K antagonists, heparin) with their high inter-individual variability on dose response. Coagulatory activity is most commonly assessed by either prothrombin time (PT) or activated partial thromboplastin time (aPTT). Both of these biomarkers are routinely used clinically, not only to monitor drug therapy, but also as diagnostic tests for bleeding disorders.

PT is generally expressed as international normalized ratio (INR) to allow for comparison of results across laboratories and test kits (Marshall et al., 1987; Palareti et al., 1987). The INR indirectly measures the activity of the extrinsic as well as common part of the coagulation network, evaluating the coagulation factors VII, X, V, II, and I (fibrinogen) (Raber, 1990), making it the first choice to monitor warfarin efficacy.

In contrast to PT and INR, aPTT is an indirect measure of the intrinsic plus common pathway, evaluating factors VIII, IX, X, XI, XII, V, II, and I (Raber, 1990).

The efficacy as well as safety of a drug may depend on demographic factors like age, sex, or body composition. In addition, genetic polymorphisms have been shown to have a strong impact on certain drugs and can account for a substantial portion of the variability between subjects treated with these compounds (Wadelius et al., 2009). For warfarin, polymorphism of the Cytochrome P4502C9 (*CYP2C9*) enzyme has been shown to lead to warfarin hypersensitivity. Two relatively common variants of *CYP2C9* with reduced enzymatic activity have been identified, CYP2C9*2 and CYP2C9*3. Patients carrying these genetic variants generally require lower maintenance doses of warfarin (Aithal et al., 1999; Higashi et al., 2002). Additionally, variations in the vitamin K epoxide reductase complex subunit 1 (*VKORC1*) gene, which encodes *VKORC1*, the key enzyme within the vitamin K cycle and target of warfarin, have been identified as contributors to the need for warfarin dose adjustments.

For the most part, anticoagulant treatment starts out with a “one dose fits all” approach, and is subsequently adjusted depending on the results of the INR or aPTT test, depending on the anticoagulant that is used (Smythe et al., 2016; Witt et al., 2018). This approach however is prone to error and can result in a high number of adverse events. Modeling and simulation (M&S) is an integrative science that combines knowledge about (patho-)physiological pathways, drug characteristics, and subject specific information into predictive mathematical models. It has been demonstrated to have wide ranging utility in drug development and regulatory review, and is used in 90% of all FDA drug approvals (Lalonde et al., 2007; Lee et al., 2011).

Quantitative systems pharmacology (QSP)—a sub-discipline of M&S—represents a powerful method, to mathematically describe complex biological systems. QSP can provide an integrated understanding of the pathology of diseases that involve multiple physiological processes. It is nowadays used not only to identify and validate new drug targets, but also to aid the understanding of existing as well as the discovery of new therapeutics (Sorger et al., 2011; Trame et al., 2016).

The aim of the present analysis was to leverage a previously developed QSP model (Wajima et al., 2009; Hartmann et al., 2016) as an exemplar of how a QSP model of the anticoagulation cascade can be modified in order to enable for the first time quantitatively the prediction of clinically important biomarkers, INR and aPTT, for commonly used anticoagulants to help optimize anticoagulant treatment for patients. In order to broaden applicability and improve predictability of the model, the effects of genetic polymorphisms for *CYP2C9* and *VKORC*1 with known impact on the PK of widely used anticoagulants were implemented.

## Material and Methods

### Patient Data

The data used for this analysis was received from the University Hospital of Schleswig-Holstein, Germany and consist of routine clinical data from 373 Caucasian adults collected during therapeutic drug monitoring (TDM). Using TDM allows for individualized adaptive anticoagulant dosing in response to the patient’s measured INR to assure optimal individual dosing to reach the best benefit-risk ratio in terms of efficacy and safety. All patients were under either steady-state warfarin/phenprocoumon or rivaroxaban treatment for prophylactic VTE therapy. In Germany, the standard treatment when VKA therapy is indicated comprises phenprocoumon; however—due to its shorter half-life—warfarin is given if patients are at heightened risk of bleeding. For the patients treated with phenprocoumon, the respective dosing amount was converted into the corresponding warfarin dosing (3 mg phenprocoumon equals to 5 mg warfarin) to facilitate the use in the model, as described previously (Crespi and Miller, 1997). Hereafter, all patients treated with either warfarin or phenprocoumon in our data are referred to being under warfarin therapy. The data used for this analysis included INR measurements of 155 patients on warfarin (n = 118 on phenprocoumon), as well as aPTT measurements of 218 patients on rivaroxaban treatment. The average value across all patients and dose groups was 2.4 (range: 0.99–4.4) for INR and 45.5 seconds (range: 23–67 seconds) for aPTT. Information about *CYP2C9* and *VKORC1* polymorphisms was available for all patients treated with warfarin. All patient demographics are shown in **Table 1**. The present study was performed in accordance with the ethical standards laid out in the updated relevant version of the Declaration of Helsinki, with informed signed consent having been obtained from each participant of the study or the patient’s parent or guardian.

**Table 1:** Patient Demographics.

Parameter	Values/median [range]	
	Warfarin	Rivaroxaban
N	155	218
Age, years	45.5 [17 - 78]	41.3 [17 – 72]
Weight, kg	85.3 [45 - 170]	83.1 [44 – 152]
Dose, mg	2.5 mg: n=62; 5 mg: n=88; 7.5 mg: n= 5	15 mg: n=39; 20 mg: n=179
CYP2C9 polymorphism	*1: n=112; *2: n=29; *3: n=14	–
VKORC1 polymorphism	GG: n=82; GA: n=56; AA: n=17	–

For age and weight, the values represent the median and range.

### Quantitative Systems Pharmacology Model

About one-third of warfarin dose variations are caused by genetic factors alone, while the remaining two-third are caused by a combination of factors such as age, sex, and body size. The presented work focused on providing a way to directly incorporate genetic polymorphisms as part of the structure of an established QSP model, rather than adding the polymorphism effects as covariate terms as it is generally done with factors like body weight and age. The previously developed QSP model of the human coagulation network was leveraged for this analysis (Wajima et al., 2009; Hartmann et al., 2016). In short, the model consists of 56 compartments, with each compartment representing either an active or inactive coagulation factor. Changes in factor concentrations were described by ordinary differential equations (ODEs) based on a turn-over model and mass-balance principles *via* Michaelis-Menten kinetics. Complex formations, such as formation of the Xa : Va complex from factors Xa and Va, were represented by a stoichiometric equation in which the components were assumed to combine in a 1:1 ratio and the complex formation would result in removal of the participating factors from their respective compartments. The effects of different drugs were implemented by using separate dosing compartments for the drugs used in the model (warfarin, rivaroxaban). The same parameter values as reported in the previous model were used, and no additional estimation steps were performed for this analysis.

### Implementation of Genetic Polymorphisms

The different polymorphisms for *CYP2C9*, which lead to altered metabolism of warfarin, were implemented into the model by adjusting the clearance of warfarin accordingly: for the CYP2C9*2 phenotype, warfarin clearance was decreased by 30%, whereas for CYP2C9*3, clearance was decreased by 80% of the original value (Crespi and Miller, 1997; Takanashi et al., 2000). Similar to warfarin, the clearance of phenprocoumon has been shown to be decreased for the CYP2C9 variants *2 and *3, leading to lower dosage requirements in carriers of CYP2C9*2 and *3 compared to CYP2C9 wild-type subjects (Hummers-Pradier et al., 2003; Schalekamp et al., 2004). As the changes in clearance were similar between warfarin and phenprocoumon, the same adjustments in clearance were applied in the model. The G3673A mutation within *VKORC1* causes a reduction in the activity of the enzyme, which subsequently leads to less available vitamin K epoxide. Physiologically, the reduced activity would be described by lowering the rate constant for the conversion of vitamin K epoxide to vitamin K. However, due to the dynamics of the presented model this strategy did not lead to an effect that was comparable to a real-world scenario. Therefore, the alternative of limiting the concentration of vitamin K available for the system was chosen to reproduce an effect in the model that was similar to the actual effect of the polymorphism in a human being. For heterozygotes, the available vitamin K was reduced by 22%, resembling the combined effect of one fully functional allele, and one allele with a 44% less activity. The homozygous genotype was simulated by decreasing the concentration of available vitamin K by 44% (Yuan et al., 2005).

### Model Predictions

In order to calculate the INR and aPTT for each virtual patient under different treatment regimens, the ODEs were numerically solved using a stiff ODE solver in Matlab^®^, ode15s. The system was initialized under steady-state non-clotting conditions, with the assumption that all activated factors have an initial concentration of zero. To predict long-term anticoagulant therapy, routine dosing was incorporated for warfarin and rivaroxaban, with dose amount and interval set to the dosing regimens reported from the observed data. As variability could not be accurately estimated using the systems model approach, a general variability term of 20% was added on the estimated production rates of factors II, V, VII, IX, X, XI, XII, XIII, PC, and PS and to the PK parameters of warfarin and rivaroxaban, which accounted for inter-individual variability; residual unexplained variability was not added as the primary interest was to describe the true differences in INR and aPTT between patients with various polymorphisms.

For warfarin, dosing regimens of 2.5, 5, and 7.5 mg once-daily per-oral (p.o.) at steady state were simulated. For rivaroxaban, once-daily oral dosing of 15 and 20 mg were tested. These dose levels were chosen as they reflect the TDM doses in the used dataset. Bioavailability, clearance, and volume terms for both compounds were taken from Hartmann et al. (2016). A virtual population of 1,000 subjects was created for each drug and dose regimen. The demographics and distribution of the different polymorphisms within the population thereby corresponded to the demographics and percentages of polymorphisms seen in the model development dataset (i.e. CYP2C9 *1: 72%, CYP2C9 *2: 19%, CYP2C9 *3: 9%; VKORC1 GG: 53%, VKORC1 GA: 36%, VKORC1 AA: 11%). Using the virtual population and dosing regimen specified above, the model was rerun with all parameters fixed to the final estimates to obtain INR and aPTT.

In order to obtain accurate prediction of INR and aPTT, the *in vitro* tests used to obtain those measures had to be mimicked. Drawing of a blood sample of a patient under steady-state anticoagulation treatment was simulated by taking the concentrations of all clotting factors predicted by the model after 20 days of rivaroxaban or warfarin treatment (corresponding to steady-state treatment conditions), and using them as input for the subsequent *in vitro* coagulation test. Clotting time was defined as the time when the area under the fibrin concentration-time curve reached 1,500 nM/s, which is equivalent to a 30% reduction of fibrinogen in normal plasma (Wajima et al., 2009).

Simulation of INR was performed by simulating the time-to-clot after addition of 300 nM of TF. INR was calculated as shown in equation 1:

(1) $${\left(\frac{{PT}_{test}}{{PT}_{standard}}\right)}^{ISI}$$

Where PT_standard_ represents PT for normal, standard plasma, PT_test_ represents the time-to-clot as determined with the model, and ISI being the international sensitivity index, set to 1.

For the aPTT test, the initial concentrations of factors XIa and XI were set to 0.148 × XI(0) and 0.339 × XI(0), respectively, which resembled the values that were simulated after addition of 300 nM of activator for the contact system (CA) to the plasma sample during the 3-min preincubation phase (Wajima et al., 2009; Nayak et al., 2015). The remaining initial concentrations were kept at their original levels as described above.

Model performance was assessed graphically using violin plots in R (version 3.5.2) directly comparing distribution and basic statistics (boxplots) between the predicted and observed INR and aPTT values.

## Results

### Patient Data

Data from a total of 373 patients were included into the present analysis, with 155 patients receiving warfarin, and 218 being under rivaroxaban treatment. The majority of patients included in the analysis dataset had CYP2C9*1 and *VKORC1 GG* genotypes, with a smaller number of subjects carrying CYP2C9*2 and *3, and *VKORC1 GA* and *AA* genotypes. An overview of the patient dispositions in presented in **Table 1**. The average INR for warfarin was 2.4, while the average aPTT for rivaroxaban was 45.5 seconds.

### Quantitative Systems Pharmacology Model

**Figure 1:**
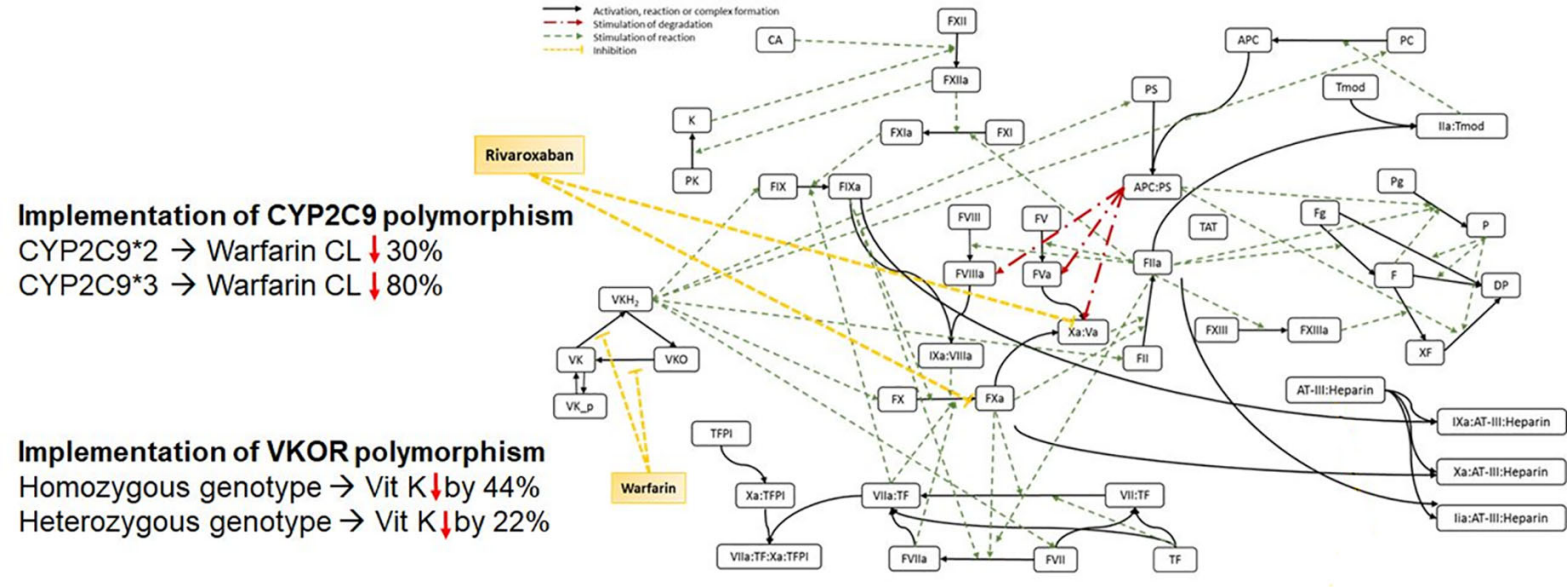
Scheme of the Quantitative Systems Pharmacology Model, including genetic polymorphisms for *CYP2C9* and *VKORC1*.

The previously described QSP model of the human coagulation network (Hartmann et al., 2016) was extended to include genetic polymorphisms of *CYP2C9* and *VKORC1* known to have an impact on the PK of commonly used anticoagulants (**Figure 1**). The effect of the polymorphisms was successfully connected to INR and aPTT outcomes to enable the prediction of these clinical important biomarkers based on patient-specific genetics.

### Prediction of INR Under Warfarin Treatment

**Figure 2:**
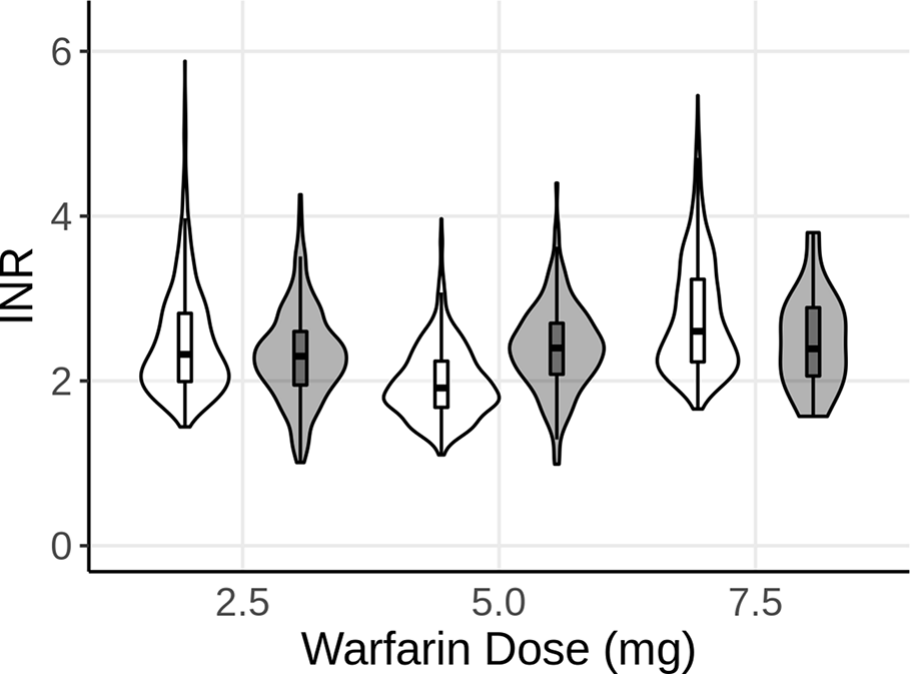
Violin plot of the predicted INR of 1,000 subjects after 2.5, 5, and 7.5 mg of steady-state warfarin (once-daily, p.o.) treatment, compared to the observed INR at the same dose levels. White represents the model predictions, while gray represents the observed data. The boxplots show the median, 25^th^, and 75^th^ percentiles (box) and 1.5 times the interquartile range (whiskers) of the simulations and observations, respectively.

The effects of genetic polymorphisms of *VKORC1* and *CYP2C9* were implemented and the model was used to predict the INR of long-term warfarin administration. For each dosing regimen (2.5, 5, and 7.5 mg warfarin once-daily, p.o.), a set of 1,000 virtual patients was simulated at steady-state. The number of patients for each combination of genetic polymorphisms for *CYP2C9* and *VKORC1* within the virtual population of each dose group was based on the ratio of polymorphisms as represented in the real-world data. Violin plots were chosen to compare the model predictions with the observed data. The violin plots shown in **Figure 2** represent the distribution of the predicted (white) and observed (gray) INR values. The overlaid boxplots show the median, 25^th^, and 75^th^ percentiles (box) with 1.5-times the interquartile range (IQR) (whiskers)of the predicted and observed INR values. As shown in **Figure 2**, the model was able to accurately predict the INR for all three dosing regimens, influenced by the different genetic polymorphisms for each patient, as indicated by the overlap of the predictions and observations.

### Prediction of aPTT Under Rivaroxaban Treatment

**Figure 3:**
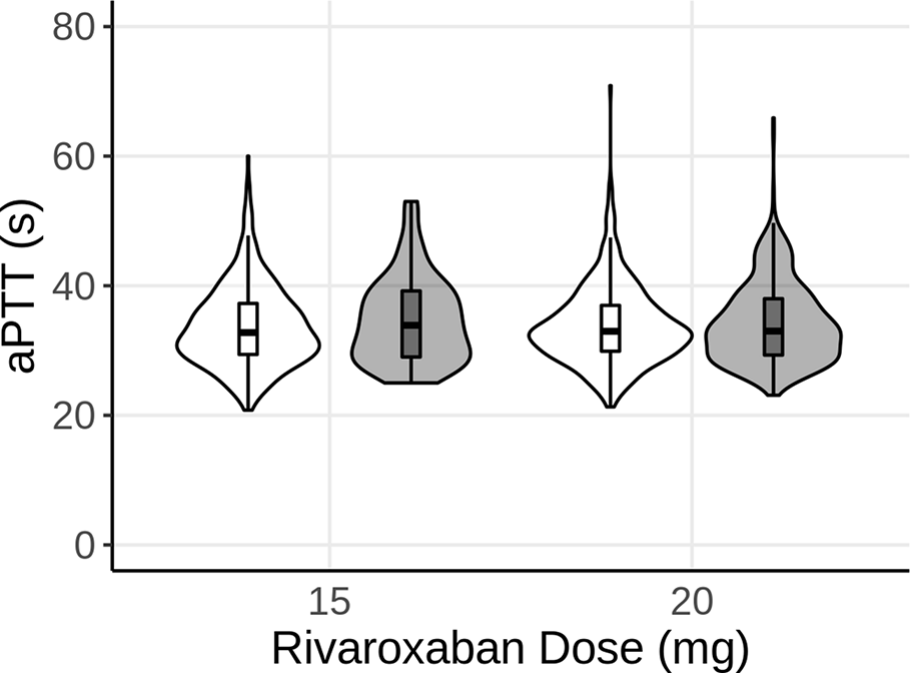
Violin plot of the predicted aPTT of 1,000 subjects after 15 and 20 mg of steady-state rivaroxaban (once-daily, p.o.) treatment. White represents the model predictions, while gray represents the observed data. The boxplots show the median, 25^th^, and 75^th^ percentiles (box) and 1.5 times the interquartile range (whiskers) of the simulations and observations, respectively.

Similar to the simulations for INR, the model was utilized to predict aPTT under steady-state rivaroxaban treatment. The dosing regimens simulated for rivaroxaban included 15 and 20mg p.o. once per day of rivaroxaban. As for the *in silico* simulations for the INR, a virtual population of 1,000 patients was created per dosing regimen of rivaroxaban. Similar to the virtual patient population for warfarin, genetic polymorphisms were simulated for each dose group based on the ratio of polymorphisms in the analysis dataset. However, as rivaroxaban is neither metabolized by either CYP2C9 or VKORC1, the polymorphisms were not expected to have any noticeable effect on the PK of rivaroxaban. Similar to **Figure 2**, **Figure 3** shows the distribution (violin) and median, 25^th^, and 75^th^ percentiles (boxplot) of the predicted (white) and observed (gray) aPTTs. The model was able to predict aPTT under rivaroxaban treatment adequately, with a good overlap between the observed values and the model predictions for the 15 and 20 mg dose groups (**Figure 3**).

## Discussion

A QSP model of the human coagulation network as described previously (Hartmann et al., 2016) was leveraged to predict clinically important outcome biomarkers for anticoagulant efficacy and safety for patients under various treatment regimens on warfarin/phenprocoumon and rivaroxaban. While mathematical models for INR and aPTT have been described before (Kogan et al., 2001; Wajima et al., 2009; Nayak et al., 2015), this analysis demonstrated for the first time the capability of such a model to adequately predict patient data from routine TDM, taking common relevant genetic polymorphisms into account.

The model was able to accurately predict INR and aPTT or patients with different *VKORC1* and *CYP2C9* phenotypes under long-term warfarin and rivaroxaban therapy. INR and aPTT are important clinical biomarkers that are frequently used in clinics to assess efficacy and safety of anticoagulants. Based on the results of these tests, dosing can be adjusted to provide the best possible outcome with the least number of adverse events, such as major bleeds. Determination of PT/INR is the method of choice to evaluate warfarin efficacy. Nevertheless, finding the correct dosing regimen for a patient receiving warfarin remains challenging due to its narrow therapeutic window and the high variability in dose response between patients. This variability is dependent on many factors, including polymorphisms of certain genes. Two genes of significant relevance for the variability observed in warfarin patients are *CYP2C9*, a warfarin metabolism enzyme, and *VKORC1*, the target enzyme of warfarin (Aithal et al., 1999; Higashi et al., 2002; Yuan et al., 2005; Nowak-Gttl et al., 2016). Patients possessing the CYP2C9*2 or CYP2C9*3 variant alleles are thought to require significantly lower mean warfarin doses. Genetic variants of *VKORC1* and *CYP2C9* have been shown to explain one third of the inter-individual variation in warfarin dose (D’Andrea et al., 2005; Puehringer et al., 2010). The prevalence of polymorphisms of *CYP2C9* and *VKORC1* varies between races and ethnicities; for instance, CYP2C9*2 and CYP2C9*3 polymorphisms are most frequently found in the Caucasian population, but are rare in Asian populations (Kimura et al., 1998; Xie et al., 2002; Kirchheiner and Brockmöller, 2005).

Algorithms and mathematical models incorporating genetic as well as demographic and clinical factors to estimate anticoagulant dosage in general and warfarin dosage in particular could potentially minimize the risk of over dosing, especially during the induction phase. Information about polymorphisms of *CYP2C9* and *VKORC1* were implemented into a previously developed QSP model (Hartmann et al., 2016), and the model was subsequently used to successfully predict INR for routinely monitored patients receiving warfarin (**Figure 2**). While the model predictions overall agree with the observed INR values, the upper range of predicted INR values, especially for the 2.5 and 7.5 mg doses, exceeds the observed values considerably. One explanation might be that the model currently does not account for other factors known to influence variability such as body weight, which could lead to higher INR values than observed for heavier subjects. Furthermore, the model provided an adequate fit for aPTT of patients receiving long-term rivaroxaban therapy (**Figure 3**). Of note, for the 15 mg group, a considerable portion of the patients had observed aPTT values around 25 s, the lowest aPTT value in the analysis dataset for this dose group, which lead to the wider lower boundary in the distribution shape of this dose group compared to the observations in the other dose group as well as the simulations. As no limits were applied to the simulations regarding possible outcomes for aPTT, the model predicted some virtual patients to have aPTT values below the lowest value observed, leading to a difference in the tails of the distributions between observed and simulated. All observed data stemmed from patients receiving long-term warfarin or rivaroxaban treatment, with dose adjustments occurring to keep the patients INR and aPTT within the desired target range (i.e. INR is usually aimed to be between 2.2 and 3.2), which would explain why no aPTT values below 23 s were observed. While it has been shown that aPTT is generally not a reliable measure of rivaroxaban efficacy (Garcia et al., 2013; Helin et al., 2013), the purpose of this analysis was to demonstrate the capability of the model to predict aPTT for a given drug, independent of whether the test provides adequate evaluation of efficacy of that drug.

As the data used for model development stemmed from TDM in clinical practice, where adaptive dosing was applied, it is expected that the variability should be reduced compared to fixed dosing. However, the model (as well as the observed data) represent data at steady-state, after the patients had been fully adjusted to an appropriate dosing regimen, which makes the degree of variability more comparable to a fixed dosing regimen.

The presented analysis does have certain limitations: While genetics account for a major portion of variability in dose response seen for certain anticoagulants, especially warfarin, they are not solely responsible. Demographic factors such as age and weight do play a significant role (Kamali et al., 2004; Miao et al., 2007), as do clinical factors such as comorbidities and co-medications (Herman et al., 2006; Limdi et al., 2009). Although the present model predicts the observed INR and aPTT measurements well by accounting for the genetic factors, the accuracy of the individual predictions could be significantly improved if additional covariates such as age, bodyweight, or co-medications could be implemented into the model. These factors would also play an important role in describing the source of variability more accurately, which in the current model is accounted for by the addition of a general variability term of 20%.

The QSP model describes the entire coagulation network and can therefore be adjusted to incorporate additional anticoagulants or compounds within the early stage of drug development. This QSP model of the anticoagulation network is the first one to date leveraged to predict clinically relevant biomarkers such as PT, INR, and aPTT as well as individual clotting factor levels within the coagulation network under different anticoagulant treatments by taking into consideration key genetic polymorphisms known to influence the efficacy and safety profile of anticoagulants. Furthermore, as demonstrated by others, a model like the one presented here can give valuable insight into the mechanisms of other disorders such as hemophilia A/B or factor VIII deficiency (Wajima et al., 2009; Nayak et al., 2015).

The presented model is an exemplar of how a QSP model can represent a useful tool to help understand and evaluate drug effects and find new targets during drug development of anticoagulants. Using a QSP-model such as the one described, the impact of known genetic polymorphisms on novel drug candidates could be predicted, subsequently supporting clinical trial optimization and dose selection, and thereby ultimately help bring novel drugs faster to the market.

## Data Availability Statement

The raw data supporting the conclusions of this article will be made available by the authors upon request, without undue reservation.

## Ethics Statement

The underlying multicenter cohort study was approved by the medical ethics committee of the University of Münster & Kiel [B304/16], Germany and written informed consent was provided in all cases prior to study participation.

## Author Contributions

SH, KB, and MT have performed the analysis. MT, UN-G, LL, SH, and KB have planned the analysis and jointly wrote the manuscript.

## Conflict of Interest

The authors declare that the research was conducted in the absence of any commercial or financial relationships that could be construed as a potential conflict of interest.
